# Supplementary material for: Influence of Sintering Parameters on Spectroscopic Properties of BMW: Eu3+ Ceramic Materials Prepared by HPLT Technique
Source: Materials (Basel). 2022 Oct 22;15(21):7410. doi: 10.3390/ma15217410 (PMC9655517; doi:10.3390/ma15217410)
Supplement: Supplementary file 1 [file materials-15-07410-s001.zip › materials-1951621-supplementary.pdf]

Supplementary Materials for

Influence of sintering parameters on spectroscopic properties of BMW:Eu^{3+} ceramic materials prepared by HPLT technique

N. Miniajluk-Gaweł*, R. Tomala, B. Bondzior, P. J. Dereń

Institute of Low Temperature and Structure Research, Polish Academy of Science, Okólna 2, 50-422 Wroclaw, Poland

**Correspondence: n.miniajluk@intibs.pl*

Table S1. The percentage distribution of the elements on the BMW:Eu-CST ceramics surface.

a. 8GPa 700ºC/time of sintering 1 min/600ºC/min

Wt% of elements	P. 1	P.2	P.3	P.4	P.5	P.6	P.7	P.8
O	16.79	16.76	14.32	11.46	11.65	11.85	13.52	11.08
Mg	32.85	35.36	17.30	05.23	05.05	05.44	07.74	03.19
Ba	27.41	27.67	37.55	29.99	37.12	32.92	66.82	40.03
Eu	02.92	03.29	03.33	29.38	17.52	23.65	04.79	04.07
W	20.02	16.92	27.48	23.94	28.66	26.15	07.13	41.64

b. 8GPa 700ºC/ time of sintering 3 min/600ºC/min

Wt% of elements	P. 1	P.2	P.3	P.4	P.5	P.6	P.7	P.8
O	14.26	12.67	12.20	10.84	12.11	13.53	11.46	10.18
Mg	20.44	19.93	15.11	04.56	04.58	02.54	02.55	02.59
Ba	35.60	36.46	40.58	35.49	37.53	71.30	37.75	37.73
Eu	03.76	03.80	04.31	19.96	18.33	04.63	04.09	04.91
W	25.95	27.14	27.81	29.15	27.45	08.00	44.14	44.58

c. 8GPa 700ºC/ time of sintering 5 min/600ºC/min

Wt% of elements	P. 1	P.2	P.3	P.4	P.5	P.6	P.7	P.8
O	23.56	22.62	21.32	21.33	19.22	22.66	23.07	23.07
Mg	35.24	20.90	26.94	04.69	07.82	02.59	11.00	02.19
Ba	27.16	37.51	30.98	36.53	31.35	66.77	59.71	65.39
Eu	01.01	00.82	03.52	19.96	22.06	01.40	01.23	01.65
W	13.03	18.15	17.24	17.48	19.55	06.58	05.00	07.70

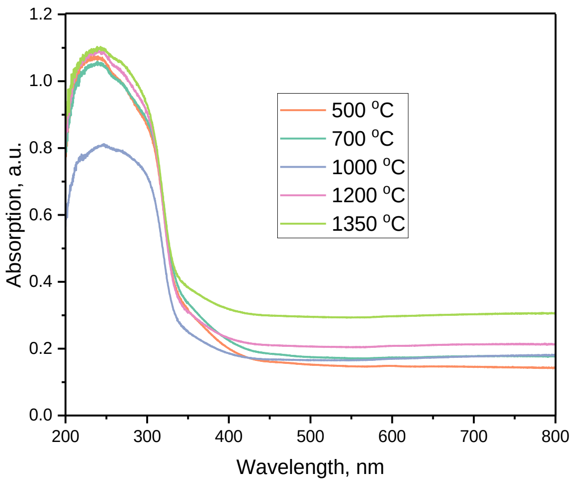

Figure S1. The absorption spectra of BMW:Eu-CT ceramic materials for which the temperature parameter was changed during the sintering process.

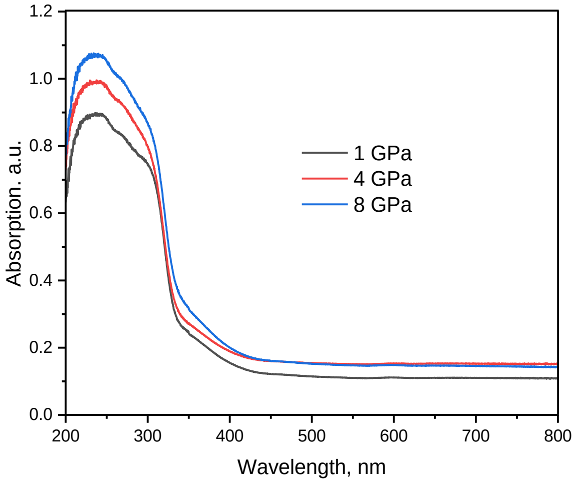

Figure S2. The absorption spectra of BMW:Eu-CP ceramic materials for which the pressure parameter was changed during the sintering process.

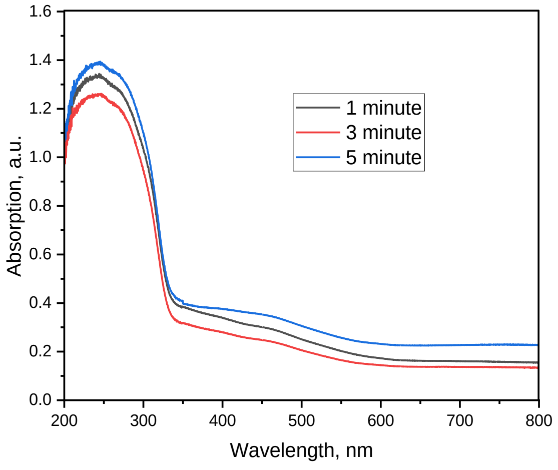

Figure S3. The absorption spectra of BMW:Eu-CTS ceramic materials for which the time of sintering parameter was changed during the sintering process.

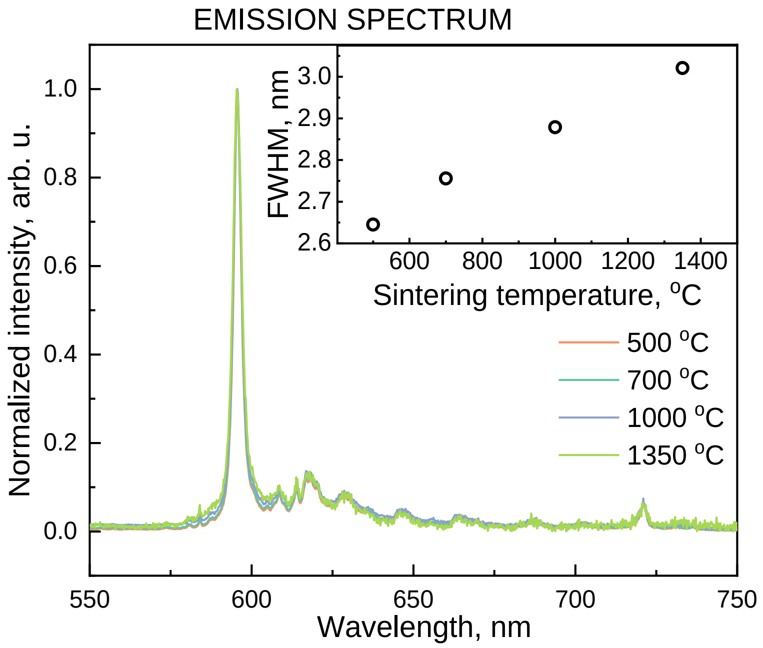

Figure S4. The emission spectra of BMW:Eu-CT ceramic materials for which the temperature parameter was changed during the sintering process.

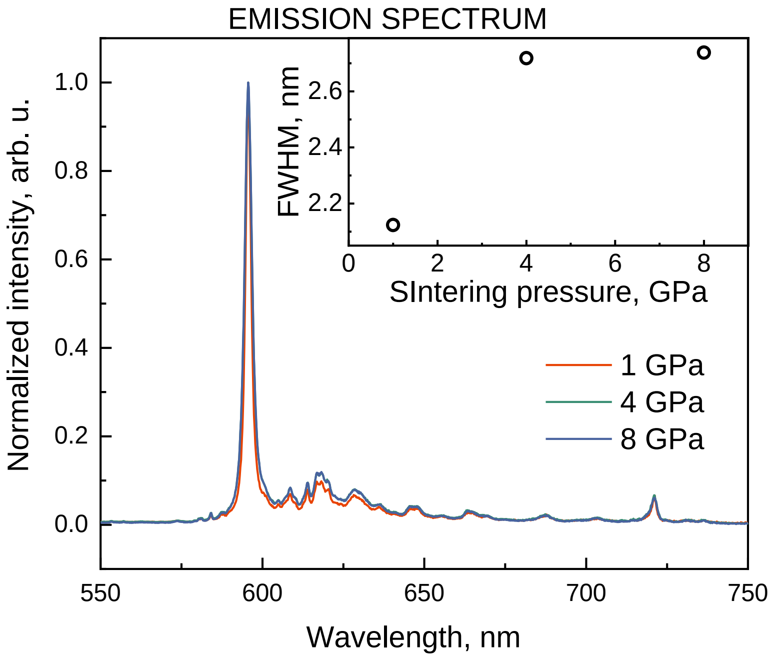

Figure S5. The emission spectra of BMW:Eu-CP ceramic materials for which the pressure parameter was changed during the sintering process.

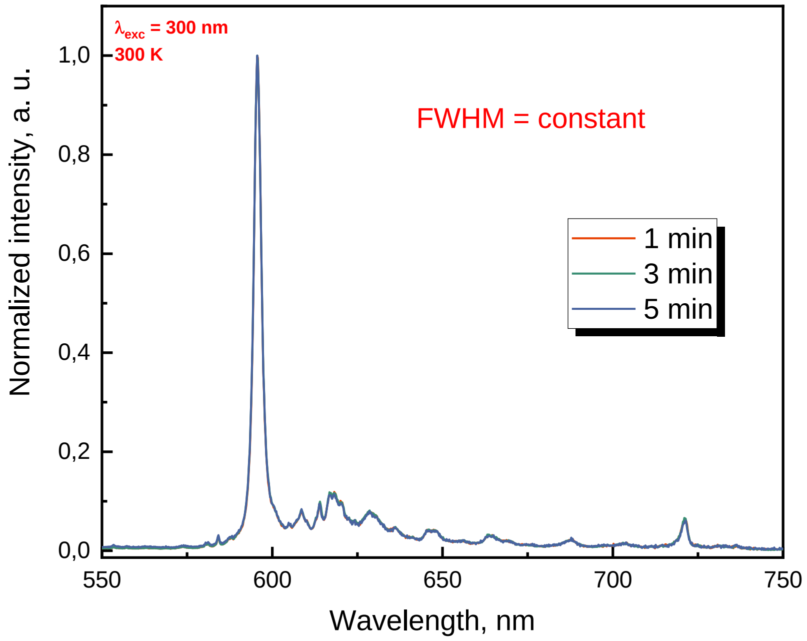

Figure S6. The emission spectra of BMW:Eu-CST ceramic materials for which the time of sintering parameter was changed during the sintering process.

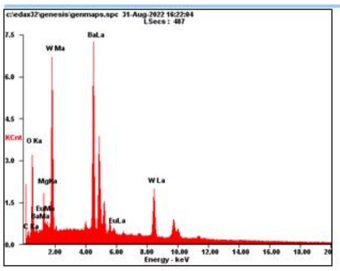

Element	Wt%	At%
CK	02.32	09.95
OK	16.21	52.24
MgK	05.69	12.07
BaL	47.02	17.65
EuL	00.47	00.16
WL	28.29	07.93
Matrix	Correction	ZAF

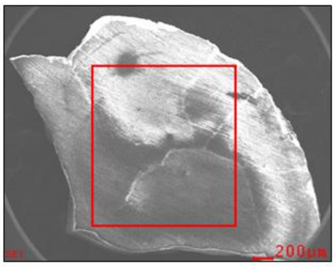

Figure S7. The SEM-EDS analysis of elements of BMW:Eu ceramic annealed at 1350 °C.

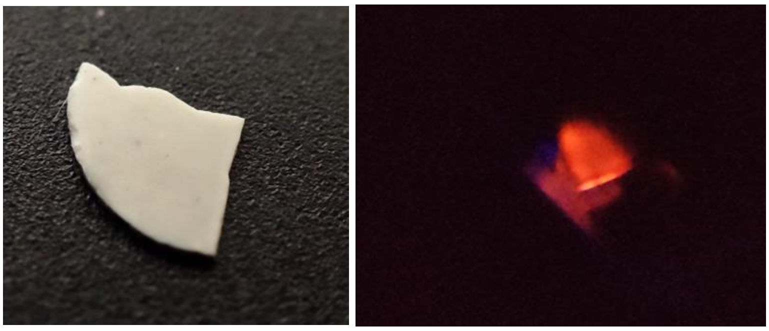

Figure S8. The photo of BMW:Eu ceramic obtained at 8 GPa and 500°C (left) and photo of emission of ceramic in transmittance setup, irradiated by 266 nm laser diode (right).
